# *Pneumocystis jirovecii* Pneumonia and Human Immunodeficiency Virus Co-Infection in Western Iran

**Published:** 2019-11

**Authors:** Arezoo BOZORGOMID, Yazdan HAMZAVI, Sahar HEIDARI KHAYAT, Behzad MAHDAVIAN, Homayoon BASHIRI

**Affiliations:** 1. Infectious Diseases Research Center, Kermanshah University of Medical Sciences, Kermanshah, Iran; 2. Department of Medical Parasitology and Mycology, School of Medicine, Kermanshah University of Medical Sciences, Kermanshah, Iran; 3. Student Research Committee, Kermanshah University of Medical Sciences, Kermanshah, Iran; 4. Department of Infectious Diseases, School of Medicine, Kermanshah University of Medical Sciences, Kermanshah, Iran

**Keywords:** *Pneumocystis jirovecii* pneumonia, Prevalence, HIV, Iran

## Abstract

**Background:**

The *human immunodeficiency virus* (*HIV*) is one of the greatest health challenges facing worldwide. The virus suppresses the immune system of the patient. The purpose of this study was to describe the epidemiology of *Pneumocystis jirovecii* colonization, rarely found in normal people, in patients with stage 4 HIV infection in Kermanshah, Iran, from Mar 1995 to Feb 2016.

**Methods:**

In this retrospective study, we surveyed medical records of stage 4 HIV-positive patients with *Pneumocystis* admitted to *Behavioral Counseling Center of Kermanshah*. Several parameters were analyzed including demographic characteristics, body mass index (BMI), treatment regimen, diagnostic methods, presenting signs and symptoms, presence of co-pathogens (bacteria, viruses, or fungi), and nadir of CD4 T-cell count before and after treatment.

**Results:**

During the study period, 114 HIV-positive patients were analyzed, of whom 93 were male and 21 were female, respectively. Of 114 cases, 26 (22.8%) patients had *Pneumocystis*. All 26 colonized patients had CD4 cell counts below 200 cells/mm3 (range 9–186). The median CD4 count increased from 91 cells/mm^3^ pre-trimethoprim/sulfamethoxazole (TMP/SMX) to an estimated 263 cells/mm^3^ after starting (TMP/SMX). BMI was normal in the majority of the patients (85%) and coughs, sputum, and chest pain (19; 73%) followed by dyspnea, weakness, and lethargy (7; 27%) were the most common presentations of fungal pneumonia.

**Conclusion:**

HIV/AIDS-infected patients are an environmental reservoir of *P. jirovecii* infection that might transmit the infection from one person to another via the airborne route. In addition, rapid identification of such individuals may reduce the morbidity and mortality rate of this disease.

## Introduction

Despite the advent of combination antiretroviral therapy (cART), about 1.5 million people die from human immunodeficiency virus (HIV) annually (1). By 2014, about 28,000 Iranians were identified to have HIV infection of whom 3300 (12.63%) lived in Kermanshah Province, west of Iran (2). Based on the WHO clinical classification system, HIV-infected patients are classified into four clinical stages from stage 1 (asymptomatic) to stage 4 (acquired immunodeficiency syndrome, AIDS) (3). The main feature of stage 4 HIV infection is severe opportunistic infections such as pneumocystosis, cryptococcosis, and candidacies (4).

*Pneumocystis jirovecii* pneumonia (PJP) is a respiratory disease caused by a yeast-like fungus. Five species of *Pneumocystis* have been identified: *P. carinii* and *P. wakefieldiae* in rats, *P. jirovecii* in humans, *P. murina* in mice, and *P. oryctolagi* in rabbits (5). PJP usually occurs in individuals with compromised immune systems such as malnourished infants, patients receiving drugs for organ transplantation, cancer patients undergoing chemotherapy, or HIV/AIDS patients. The disease is also rarely found in normal people (6).

The first clinical cases of PJP were documented during the Second World War in Europe in 1942 (7). The first PJP infected case in Iran was identified in an orphanage and confirmed by autopsy in 1964 (8). These studies were continued by Kohout et al in Shiraz, Iran. They compared the immunoglobulin levels in PJP and non- PJP patients from 1966 to 1968 (9). However, *Pneumocystis* pneumonia is still a rare disease. The first case of this disease in Kermanshah Province was reported in a patient with HIV infection in 2003. The patient died several weeks after *Pneumocystis* was diagnosed and treated (10).

Since clinical signs are not specific for a diagnosis of PJP, clinicians should be aware of this co-infection in the differential diagnosis of HIV opportunistic infections. Any delay in diagnosis and treatment may result in longer hospitalization and increased morbidity and mortality. Although many studies have evaluated the prevalence of the HIV infection in Iran, few data are available about opportunistic infections such as *P. jirovecii* in HIV patients. The primary aim of this study was to determine the prevalence of *P. jirovecii* in patients with stage 4 HIV infection in Kermanshah, Iran.

## Materials and Methods

The study protocol was approved by the Ethics Committee of Kermanshah University of Medical Science and ethical clearance was obtained.

The medical records of all HIV-infected patients diagnosed with WHO stage 4 conditions admitted to *Behavioral Counseling Center of Kermanshah*, Iran, from Mar 1995 to Feb 2016 were retrospectively reviewed. Behavioral Diseases Counseling Center has a free-of-charge care center to provide medical care, counseling, and drugs for HIV-infected patients and injection drug users. Data collected from the patients’ medical records included *P. jirovecii* infection, demographic characteristics, treatment regimen, diagnostic methods, presenting signs and symptoms, presence of copathogens (bacteria, viruses, or fungi), and nadir of CD4 T-cell count before and after treatment. In addition, the body mass index (BMI) was calculated by dividing body weight (in kilograms) by the square of height (in meters). Statistical analysis was performed using SPSS 13 (ver. 13.5; Inc, Chicago, IL, USA).

## Results

During the study period, 114 HIV-infected patients with WHO clinical stage 4 conditions were admitted to Behavioral Counseling Center of Kermanshah, Iran, of whom 93 (81.6%) were male and 21 (18.4%) were female.

Twenty-six HIV patients were diagnosed with PJP infection. The median age of the patients was 42.69 yr (range: 14 yr to 69 yr) and the male/female ratio was 4.2 (21/5). Cough, sputum, and chest pain (19; 73%) followed by dyspnea, weakness, and lethargy (7; 27%) were the most common presentations of fungal pneumonia.

A BMI of 18.5 to 24.9 is considered healthy, while a value below 18.5 indicates malnutrition. The BMI range of the patients was 12.1 to 24.4 (mean: 20.40) and only four patients had a BMI below 18.5.

**Fig. 1:**
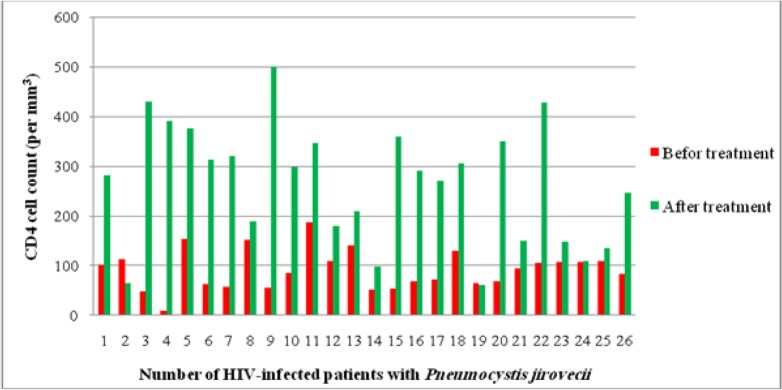
Nadir of CD4 T-cell count before and after treatment in stage 4 HIV-positive patients with *Pneumocystis jirovecii*

All 26 colonized patients had CD4 cell counts below 200 cells/mm3 (mean± SD: 91.81± 39.76, range: 9–186) (normal range=500–1200 cells/mm3). All of the patients received trimethoprim-sulfamethoxazole (co-trimoxazole) as the first-choice treatment and no side effects were reported. Except for two cases (9%), the CD4+ T-cell count increased in HIV patients with *P. jirovecii* infection after the initiation of co-trimoxazole therapy (mean± SD: 263.58± 12.70, range: 60–500). Regarding concomitant co-existing infections, 12 patients did not have any co-infections, 7 patients had a combination of various infections including TB, toxoplasmosis, retinitis, hepatitis C, etc. TB and toxoplasmosis alone were detected in 7 (33.3%) and 2 (16.6%) cases, respectively (Fig. 1). A diagnosis of PJP was made by microbiological staining of bronchoalveolar lavage (BAL) specimens in one (3.86%) patient, High Resolution Computed Tomography (HRCT) in 8 (30.76%) patients, and chest radiology in 17 (65.38%) cases.

## Discussion

Opportunistic infections (OIs) are one of the identified causes of increased immunodeficiency in patients with HIV (11). There are many reports on the co-infection of *P. jirovecii* with other pathogens such as bacteria, fungi, or protozoa in patients infected with HIV/AIDS. The results of the current retrospective study showed that more immunocompromised patients with PJP had TB infection, which was similar to a recent study in Iran (12). CD4^+^ T cells and MHC class II molecules that induce CD4^+^ T cell responses are critical for control of Mycobacterium tuberculosis infection. Therefore, TB infection is both the most common AIDS-indicator disease and has an immunosuppressive effect. Hence, when one opportunistic infection is diagnosed in an HIV infected patient, the physician should suspect other co-infections that are common in HIV/AIDS patients.

The prevalence of PJP infection in HIV infected patients (stage 4) was 22.8%, which was similar to a previous study in India (13). However, lower than studies conducted in Brazil (14), Ahvaz, Iran (12) and Tehran, Iran, (15) in which the prevalence of PJP ranged from 27% to 40% depending on the study population and diagnostic technique. However, many investigations has shown higher sensitivity of PCR-based assays compared to direct examination because it allows detection of low levels of microorganisms in the sample (16,17).

In the current study, although the odds of PJP infection were 4 times as high in men infected with HIV/AIDS than women, this variable had no significant effect on the development of this disease (*P*=0.904). These findings are similar to the results of a recent study in Pakistan (18) but they are inconsistent with the results of a review study indicating that the acquisition of PJP is related to gender, race or ethnicity, and HIV co-infection (19).

In our study, all patients had CD4 cell counts below 200 cells/mm^3^ (range: 9–186). The most significant risk factor for the development of PJP in HIV/AIDS infected patients is low lymphocyte counts, which is consistent with the results of previous studies (20, 21).

Regarding PJP treatment, all HIV/AIDS patients in the present study, except for two patients (9%), received TMP-SMX as the first-line treatment. The main predictor of failure was poor CD4 cell recovery or no increase in the CD4 count after TMP/SMX. Castro et al reported that failure of PJP treatment with TMP/SMX occurs in up to 20% of the patients (22). *P. jirovecii* cannot be cultured; therefore, no data on drug resistance is available. However, point mutations in the dihydropteroate synthetase (DHPS) gene are associated with TMP-SMX resistance (23).

Although opportunistic infections such as *P. jirovecii* have been reported in malnourished individuals (24, 25), the present study showed that the majority of patients with PJP infection (84.6%) had a normal BMI, as previously reported by other studies (26, 27). However, this finding needs to be evaluated in large randomized trials.

The present study had some limitations. Due to the retrospective nature of the study, we could not evaluate laboratory test results such as the serum level of lactate dehydrogenase, C-reactive protein, and albumin, which could influence the results of PJP.

## Conclusion

We found a relatively high prevalence of PJP infection in HIV/AIDS patients in Kermanshah Province. Rapid identification of such individuals may reduce the morbidity and mortality of this disease. Furthermore, molecular techniques are applied as a sensitive method to identify PJP in biological samples and to prevent unnecessary exposure to TMP-SMX. However, further studies are needed for better comprehension of the epidemiology of *Pneumocystis* in the area under study.

## Ethical considerations

Ethical issues (Including plagiarism, informed consent, misconduct, data fabrication and/or falsification, double publication and/or submission, redundancy, etc.) have been completely observed by the authors.

## References

[1] Chang CC Crane M Zhou J (2013). HIV and co-infections. Immunol Rev, 254( 1): 114–42. 23772618 10.1111/imr.12063 PMC3697435

[2] Khademi N Reshadat S Zangeneh A (2017). A comparative study of the spatial distribution of HIV prevalence in the metropolis of Kermanshah, Iran, in 1996–2014 using geographical information systems. HIV Med, 18( 3): 220–4. 27535117 10.1111/hiv.12416

[3] World Health Organization Consolidated guidelines on the use of antiretroviral drugs for treating and preventing HIV infection: recommendations for a public health approach . Geneva : World Health Organization ; 2016 . http://www.who.int/hiv/pub/arv/arv-2016/en/ 27466667

[4] Limper AH Adenis A Le T (2017). Fungal infections in HIV/AIDS. Lancet Infect Dis, 17( 11): e334–e343. 28774701 10.1016/S1473-3099(17)30303-1

[5] Aliouat-Denis C-M Chabé M Demanche C (2008). *Pneumocystis* species, co-evolution and pathogenic power. Infect Genet Evol, 8( 5): 708–26. 18565802 10.1016/j.meegid.2008.05.001

[6] Hof H (2012). *Pneumocystis jirovecii*: A peculiar fungus posing particular problems for therapy and prophylaxis. Mycoses, 55( s1): 1–7.

[7] Van der Meer G Brug SL (1942). Infection à Pneumocystis chez l’homme et chez les animaux. Ann Soc Belge Med Trop, 22: 301.

[8] Post C Dutz W Nasarian I (1964). Endemic Pneumocystis carinii pneumonia in south Iran. Arch Dis Child, 39( 203): 35–40. 14160085 10.1136/adc.39.203.35 PMC2019161

[9] Kohout E Post C Azadeh B (1972). Immunoglobulin levels in infantile pneumocystosis. J Clin Pathol, 25( 2): 135–140. 4536973 10.1136/jcp.25.2.135 PMC477243

[10] Janbakhsh A Sayad B Mikaili A (2003). The first report of *Pneumocystis carini* pneumonia in a patient with HIV infection in Kermanshah. J Kermanshah Univ Med Sci, 7( 2): 60–65.

[11] Nazari N Bozorgomid A Janbakhsh A (2018). *Toxoplasma gondii* and human immunodeficiency virus co-infection in western Iran: A cross sectional study. Asian Pac J Trop Med, 11( 1): 58–62.

[12] Aboualigalehdari E Mahmoudabadi AZ Fatahinia M (2015). The prevalence of *Pneumocystis jirovecii* among patients with different chronic pulmonary disorders in Ahvaz, Iran. Iran J Microbiol, 7( 6): 333–7. 26885334 PMC4752688

[13] Kaur R Mehra B Dhakad MS (2017). Fungal Opportunistic Pneumonias in HIV/AIDS Patients: An Indian Tertiary Care Experience. J Clin Diagn Res, 11 ( 2 ): DC14 – DC19 . 10.7860/JCDR/2017/24219.9277 PMC5376915 28384860

[14] Pereira RM Müller AL Zimerman RA (2014). High prevalence of *Pneumocystis jirovecii* colonization among HIV-positive patients in southern Brazil. Med Mycol, 52( 8): 804–9. 25288653 10.1093/mmy/myu059

[15] Homayouni MM Behniafar H Mehbod ASA (2017). Prevalence of *Pneumocystis jirovecii* among immunocompromised patients in hospitals of Tehran city, Iran. J Parasit Dis, 41( 3): 850–53. 28848290 10.1007/s12639-017-0901-y PMC5555945

[16] Wang D Hu Y Li T (2017). Diagnosis of *Pneumocystis jirovecii* pneumonia with serum cell-free DNA in non-HIV-infected immunocompromised patients. Oncotarget, 8( 42): 71946–53. 29069759 10.18632/oncotarget.18037 PMC5641102

[17] Khodavaisy S Mortaz E Mohammadi F (2015). *Pneumocystis jirovecii* colonization in Chronic Obstructive Pulmonary Disease (COPD). Curr Med Mycol, 1( 1): 42–8. 28680980 10.18869/acadpub.cmm.1.1.42 PMC5490321

[18] Zubairi ABS Shahzad H Zafar A (2016). Clinical outcomes of *Pneumocystis* pneumonia from a tertiary care centre in Pakistan. J Pak Med Assoc, 66( 11): 1367–71. 27812050

[19] Miller RF Huang L Walzer PD (2013). *Pneumocystis* pneumonia associated with human immunodeficiency virus. Clin Chest Med, 34( 2): 229–41. 23702173 10.1016/j.ccm.2013.02.001

[20] Javier B Susana L Santiago G (2012). Pulmonary coinfection by *Pneumocystis jiroveci* and *Cryptococcus neoformans*. Asian Pac J Trop Biomed, 2( 1): 80–2. 23569840 10.1016/S2221-1691(11)60195-0 PMC3609199

[21] Llibre JM Revollo B Vanegas S (2013). *Pneumocystis jirovecii* pneumonia in HIV-1-infected patients in the late-HAART era in developed countries. Scand J Infect Dis, 45( 8): 635–44. 23547568 10.3109/00365548.2013.777778

[22] Castro JG Morrison-Bryant M (2010). Management of *Pneumocystis jirovecii* pneumonia in HIV infected patients: current options, challenges and future directions. HIV AIDS (Auckl), 2 : 123 – 34 . 22096390 10.2147/hiv.s7720 PMC3218692

[23] Ponce CA Chabé M George C (2017). High Prevalence of *Pneumocystis jirovecii* Dihydropteroate Synthase Gene Mutations in Patients with a First Episode of *Pneumocystis* Pneumonia in Santiago, Chile, and Clinical Response to Trimethoprim-Sulfamethoxazole Therapy. Antimicrob Agents Chemother, 61( 2): e01290–16. 27855071 10.1128/AAC.01290-16 PMC5278682

[24] Jones KD Berkley JA (2014). Severe acute malnutrition and infection. Paediatr Int Child Health, 34 Suppl 1: S1–S29. 25475887 10.1179/2046904714Z.000000000218 PMC4266374

[25] Attalla El Halabieh N Petrillo E Laviano A (2016). A Case of *Pneumocystis jirovecii* Pneumonia in a Severely Malnourished, HIV-Negative Patient: A Role for Malnutrition in Opportunistic Infections? JPEN J Parenter Enteral Nutr. 40 ( 5 ): 722 – 24 . 25172049 10.1177/0148607114548072

[26] Asai N Motojima S Ohkuni Y (2012). Non-HIV *Pneumocystis* pneumonia: do conventional community-acquired pneumonia guidelines under estimate its severity? Multidiscip Respir Med , 7 ( 1 ): 2 . 22958656 10.1186/2049-6958-7-2 PMC3415119

[27] Lee SH Huh KH Joo DJ (2017). Risk factors for *Pneumocystis jirovecii* pneumonia (PJP) in kidney transplantation recipients. Sci Rep, 7( 1): 1571. 28484270 10.1038/s41598-017-01818-w PMC5431538

